# Dynamics of stored lipids in fall migratory monarch butterflies (*Danaus plexippus*): Nectaring in northern Mexico allows recovery from droughts at higher latitudes

**DOI:** 10.1093/conphys/coad087

**Published:** 2023-11-24

**Authors:** Keith A Hobson, Orley Taylor, M Isabel Ramírez, Rogelio Carrera-Treviño, John Pleasants, Royce Bitzer, Kristen A Baum, Blanca X Mora Alvarez, Jude Kastens, Jeremy N McNeil

**Affiliations:** Department of Biology, University of Western Ontario, 1151 Richmond St, London, ON, N6A 5B7, Canada; Environment and Climate Change Canada, 11 Innovation Blvd, Saskatoon, SK, S7N 3H5, Canada; Department of Ecology and Evolutionary Biology and Kansas Biological Survey and Center for Ecological Research, University of Kansas, 1450 Jayhawk Blvd, Lawrence, KS 66045, USA; Centro de Investigaciones en Geografia Ambiental, Universidad Nacional Autónoma de Mexico, Antigua Carretera A Patzcuaro 8701, Ex hacienda San Jose de la Huerta, 58190, Morelia, Michoacán, Mexico; Facultad de Medicina Veterinaria y Zootecnia, Universidad Autónoma de Nuevo León, C. Francisco Villa 20, Escobedo, Nuevo León, México; Department of Ecology, Evolution, and Organismal Biology, 2200 Osborne Dr, Iowa State University, Ames, IA 5011, USA; Department of Plant Pathology, Entomology, and Microbiology, 2213 Pammel Dr., Iowa State University, Ames, IA 50011, USA; Department of Integrative Biology, Oklahoma State University, 501 Life Sciences E, Stillwater, OK 74078, USA; Department of Biology, University of Western Ontario, 1151 Richmond St, London, ON, N6A 5B7, Canada; Kansas Biological Survey & Center for Ecological Research, University of Kansas, 2101 Constant Ave., Lawrence, KS 66047, USA; Department of Biology, University of Western Ontario, 1151 Richmond St, London, ON, N6A 5B7, Canada

**Keywords:** Fuel, habitat conservation, lipid stores, nectar availability, wing loading

## Abstract

The eastern population of the North American monarch butterfly (*Danaus plexippus*) overwinters from November through March in the high-altitude (3000 m+) forests of central Mexico during which time they rely largely on stored lipids. These are acquired during larval development and the conversion of sugars from floral nectar by adults. We sampled fall migrant monarchs from southern Canada through the migratory route to two overwintering sites in 2019 (n = 10 locations), 2020 (n = 8 locations) and 2021 (n = 7 locations). Moderate to extreme droughts along the migratory route were expected to result in low lipid levels in overwintering monarchs but our analysis of lipid levels of monarchs collected at overwintering sites indicated that in all years most had high levels of lipids prior to winter. Clearly, a significant proportion of lipids were consistently acquired in Mexico during the last portion of the migration. Drought conditions in Oklahoma, Texas and northern Mexico in 2019 resulted in the lowest levels of lipid mass and wing loading observed in that year but with higher levels at locations southward in Mexico to the overwintering sites. Compared with 2019, lipid levels increased during the 2020 and 2021 fall migrations but were again higher during the Mexican portion of the migration than for Oklahoma and Texas samples, emphasizing a recovery of lipids as monarchs advanced toward the overwintering locations. In all 3 years, body water was highest during the Canada—USA phase of migration but then declined during the nectar foraging phase in Mexico before recovering again at the overwintering sites. The increase in mass and lipids from those in Texas to the overwintering sites in Mexico indicates that nectar availability in Mexico can compensate for poor conditions experienced further north. Our work emphasizes the need to maintain the floral and therefore nectar resources that fuel both the migration and storage of lipids throughout the entire migratory route.

## Teaser

Migrating monarch butterflies require lipids to fuel their journey and to overwinter in Mexico. We examined the dynamics of lipid storage over their journey and found that they can recover lipids in Mexico following drought conditions in the US southwest. Our findings evoke considerable conservation consequences.

## Introduction

The eastern North American breeding population of the monarch butterfly (*Danaus plexippus*) has become an iconic example of long-distance insect migration. Each year, millions of adults migrate from their natal sites in the eastern United States and Canada to favorable thermal environments at the overwintering sites in the high-altitude oyamel fir (*Abies religiosa*) forests of central Mexico (Alonso-Mejía *et al.,* 1997; Williams and Brower, 2015). Because the distances traveled to reach the overwintering sites can be greater than 3000 km, and can take at least 75 days, understanding the energy needed to sustain flight and the dynamics and efficiencies of these flights are of interest. While there has been some attention to energetics in terms of lipid acquisition and depletion in locusts (Talal *et al.,* 2023), as well as flight dynamics in the monarch (Gibo, 1986), how these processes enable migrations that cover great distances and months of flight are not well understood.

It is well established that the fall migration is fueled by nectar (Beall, 1948; Cenedella, 1971; Brown and Chippendale, 1974; Turunen and Chippendale, 1980; Gibo and McCurdy, 1993; Brower *et al.,* 2006, 2015). Interactions between weather and nectar availability, the source of the carbohydrates that are converted to lipids during migrations, and weather conditions at overwintering sites clearly are factors that deserve attention. There is also a need to establish a current energetic baseline since nectar availability could decline due to agricultural intensification and changes in climate predicted for both fall and spring migrations and the overwintering period (Oberhauser and Peterson, 2003; Nail and Oberhauser, 2015; Espeset *et al.,* 2016; Saunders *et al.,* 2019; Wilcox *et al.,* 2019).

Despite considerable interest in the conservation of migratory monarch butterflies in North America (Flockhart *et al.,* 2015; Oberhauser *et al.,* 2017; Thogmartin *et al.,* 2017a, 2017b; Malcolm, 2018; Wilcox *et al.,* 2019; Taylor *et al.,* 2020; Zylstra *et al.,* 2021) and recognition of the importance of nectar availability to their conservation (Inamine *et al.,* 2016; Agrawal and Inamine, 2018), little is known of the dynamics of lipid synthesis and storage during migrations. This stands in contrast to conservation concerns directed at the breeding (Lark *et al.,* 2015; Pleasants, 2017) and wintering grounds (Williams *et al*., 2015; Oberhauser *et al.,* 2017; Saunders *et al.,* 2019; Zylstra *et al.,* 2021). Previous research assumed that monarchs generally increase lipid stores as they approach their wintering grounds and that Texas and northeast Mexico provide the bulk of the lipids required for both overwintering and subsequent reproduction in spring (Brower, 1985; Alonso-Mejía *et al.,* 1997; but see Sánchez-Tlacuahuac *et al*., 2022). For example, Brower *et al.* (2015) showed that during the drought in Texas in 2011, lipid levels were low in locally captured monarchs but were higher in individuals collected near and at overwintering sites. In addition, Hobson *et al.* (2020) used stable isotope tracking approaches on bulk lipids to demonstrate that, at least in some years, much of the overwintering lipid stores are likely obtained throughout the floral corridor in Mexico leading to the wintering grounds (see also Pilecky *et al.,* 2023).

Given that overwintering population size may be affected, in part, by mortality during the fall migration (Ries *et al.,* 2015a, 2015b; Inamine *et al.,* 2016; Agrawal and Inamine, 2018; Kantola *et al.,* 2019; Mora-Alvarez *et al*., 2019; Tracy *et al.,* 2019; Taylor *et al.,* 2020) and/or by the condition of monarchs when they arrive at overwintering sites (Inamine *et al.,* 2016), an eco-physiological examination of interactions between weather and monarch fuel loads during the fall migration is warranted. However, interpretation of cause and effect involved in these interactions are difficult. Lipid levels are likely to be affected differently as monarchs pass through regions in which floral resources are abundant or scarce. Droughts are expected to result in low lipid levels, while monarchs advancing through the mid elevations in Mexico are expected to increase in mass and lipid levels but these ideas have not been tested. In addition, if high wing loading increases flight speed, glide ratio and overall metabolic efficiency, abundant nectar and water would appear to enable increases in migratory success (Gibo, 1993; Kvist *et al.,* 2001; but see Becker, 2008; Bowlin and Wikelski, 2008).

In this study, we examined indices of lipid and water levels together with measurements of wing loading of monarchs collected at 14 locations during the fall migration from southern Ontario (42.6°N) through to two overwintering sites (Chincua and Cerro Pelon, 19.4°N) in central Mexico. Samples were obtained during the fall of 2019, a period of moderate to extreme drought in central Texas, and during falls of 2020 and 2021 when conditions in that region improved (Drought Monitor Maps 2019–2021; https://droughtmonitor.unl.edu/). Since monarchs that reached overwintering sites represent migratory success, by comparing their body conditions with values of adults sampled along the entire migratory route, we aimed to evaluate (i) the impact of local weather conditions on lipid levels of migrants as they passed through different sites and (ii) the importance of nectar foraging during the latter portion of the migration as this appears important to their overwinter survival. Our objective was to provide a first description of monarch body condition throughout the length of fall migration and to suggest the importance of these results to monarch conservation, as well as future studies that must focus on current and predicted climate variation.

**Figure 1:**
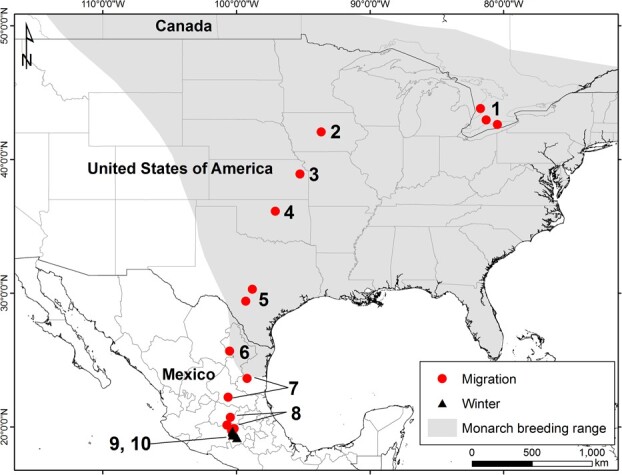
Location of collection sites for migrating monarch butterflies during the fall migration (September to December) 2019–2021. Numbers correspond to the groupings used in Figs 2-4 (Ontario, 1; Iowa, 2; Kansas, 3; Oklahoma, 4; Texas, 5; Nuevo Leon, 6, Ciudad Victoria, San Luis Potosi, 7; Queretero, Gunajuato, Contepec, 8; Sierra Chincua, 9; Cerro Pelon, 10).

## Methods

In 2019, we established a coordinated collection of fall migrant monarchs at 12 sites representing eight site clusters (groups) along the migratory route from southern Ontario to central Mexico and at two ultimate overwintering sites (i.e. Site 13, Group 9 and Site 14, Group 10; Fig 1). Six sites in central Mexico (Sites 7–12, Group 8) were only sampled during the fall of 2019. Site locations, dates of sampling, elevation and sun angle at solar noon are listed in Supplementary Table S1. Samples of nectaring and roosting migrants were obtained opportunistically, often over several days at any given site, and stored in coolers in the field until they could be frozen (−20°C). All freshly collected monarchs were weighed prior to freezing (to at least 0.001 g; Acculab Pocket Pro Series, Model #PP-2060D electronic balance or Sartorius Practum 224-1S analytical balance) to establish wet mass and again after drying to establish dry mass. Wings were measured for forewing and hind wing length (±0.1 mm) using calipers and then removed for subsequent scanning for surface area (below). Bodies were then retained for lipid extraction. Monarchs sampled at the overwintering sites in 2019 and 2020 were oven dried in Mexico for 72 hours (at 50°C) and stored in individual paper envelopes along with silica desiccant. In 2021, monarchs collected at the overwintering site were freeze dried at the Universidad Nacional Autónoma de Mexico, Morelia, Michoacán, Mexico, shortly after collection. All other samples were frozen (−20°C) shortly after collection before freeze drying (Sites 1, 2, 6) and shipping or shipped frozen (Sites 3, 4, 5) for further processing at our laboratory at Western University. Freeze drying was performed on various machines depending on location and for varying periods (8–48 hours) depending on number of samples measured and their size until constant dry mass was achieved.

To measure wing surface area, wings from each sample were detached from the body, placed without overlap in clear plastic holders (AmazonBasics model DHNB002; Seattle, WA, USA) and scanned with an optical area meter (LI-COR Inc. model LI-3100C; Lincoln, NE, USA). To ensure accuracy, samples were measured three times, and the mean used as the final measurement. Standard error for each sample was ±0.06 cm^2^.

**Figure 2:**
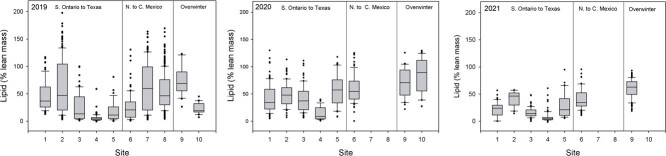
Lipid content of monarch butterflies collected during fall migration 2019–2021 expressed as a percent of lean body mass (minus wings) for collection sites as indicated in Figure 1. Box and whisker plots depict the interquartile range between the maximum and minimum whisker (quartile 3 minus quartile 1), the box depicts the position of the upper and lower 25% of the data, the median (horizontal bar in the box) and outliers (those single points beyond the interquartile range.

Lipids were removed from whole dried bodies (minus wings) by successive soaking and rinsing in a 2:1 chloroform/methanol solvent. After opening the abdominal cavity, each body was placed into a 25-ml glass scintillation vial with ~ 20 ml of solvent, capped and gently agitated before a soak of 4 hours. The solvent was then decanted into a weighed receiving vial in a fume hood and the process repeated five times and to a point when the solvent was uniformly clear. The solvent extracts were then evaporated to dryness and the vessel weighed to provide body lipid mass for each individual.

In 2021, we discovered that some (~121 of 461) dried monarch samples received had wings that had clearly soaked up some body lipid during the period of storage and transport. So, since our procedure involved removing wings prior to lipid extraction, we corrected for this small effect by deriving a correlation between wing mass and wing area for a range of non-affected wings (n = 469 across all years) and then examined the mass of affected wings. Using our calibration relationship (r^2^ = 0.76), positive residuals of mass of affected wings were then used to correct for the lipid content of the wings by adding that mass to the extracted lipid mass of the bodies.

While not distinguished in Supplementary Table S2 or subsequent analyses, the Iowa collection in 2019 and 2020 involved individuals sampled at both diurnal nectaring sites and at nocturnal roost sites. All other sites across years were composed of only diurnal collections of nectaring individuals or fresh roadkills (i.e. Monterrey only in 2021 as part of another study).

We defined wet mass as the mass of the whole monarch before drying. Dry mass was defined as the mass of the entire monarch following drying and %water was the proportional mass difference between wet and dry mass. Lipid mass was the dry mass of lipids extracted using our solvent technique. Lean body mass was monarch mass minus wings and following lipid extraction. For this reason, lipid levels as a proportion of lean body mass can exceed 100% (i.e. lipid mass can exceed lean body mass). Wing loading was wet mass divided by wing surface area obtained from scanning. As a proxy for local nectar productivity anomaly, the Normalized Difference Vegetation Index (NDVI) Z-scores were computed using 11 years (2012–2022) of VIIRS multi-day composite NDVI time series imagery developed by USGS and distributed through https://dds.cr.usgs.gov. A complete description of NDVI methods is provided in the Supplementary Materials.

**Figure 3:**
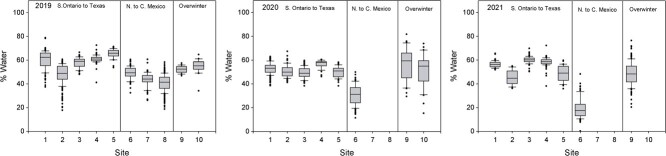
Water content of monarch butterflies collected during fall migration 2019–2021 based on difference between wet and dry weight. Collection sites as indicated in Figure 1. Box and whisker plot description given in Legend of Figure 2.

**Figure 4:**
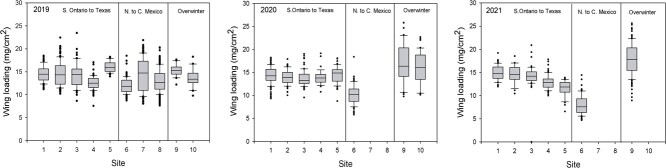
Wing loading of migrating fall monarchs collected in 2019–2021 at locations shown in Figure 1. Wing loading based on total wing area (i.e. all four wings) per individual and lean body mass. Collection sites as indicated in Figure 1. Box and whisker plot description given in Legend of Figure 2.

For our statistical analyses, we first considered each year separately and used analysis of variance (ANOVA) to compare various physiological metrics (e.g. lipid content, wing loading, water content) between sexes, sampling sites and regions. A general linear model (GLM) was used to investigate interactions between sex and location. In order to best interpret data depicted as three regions in Figs 2–4, we divided our sites into those from Ontario to Texas (Group 1), Sierra Leone to the overwintering sites (Group 2) and overwintering sites (Group 3) because these three regions were of the most biological interest. Analyses were performed using SPSS V. 24. Details of the statistical treatment for NDVI are described in Supplementary Material where three site elevation groups were defined (Groups A, B, C) for NDVI summary.

## Results

There was considerable variation among sites and years (Fig 2–4) in percent lipid, percent water and wing loading measured in fall migrating monarchs. For both sexes, we also summarized key (morphometric and physiological) parameters for each collection site for each year (Supplementary Table S2). Summaries of percent lipid, percent water and wing loading were analysed using three groups in Table 1).

**Table 1:** Summary statistics by year of changes in %lipid, %water and wing loading. Number of sites and monarch sample sizes given in Supplementary Table S1

Metric	Migration section	Sex	Year					
			2019		2020		2021	
			F (df)	*P*	F (df)	*P*	F (df)	*P*
%Lipid	S. Ontario to Texas	M	28.9 (4,249)	<0.001	9.08 (4,199)	<0.001	16.91 (4,160)	<0.001
		F	38.70 (4,195)	<0.001	15.55 (4,127)	<0.001	23.27 (4,102)	<0.001
	N to central Mexico	M	11.76 (2,252)	<0.001	NA	NA	NA	NA
		F	28.85 (2,200)	<0.001	NA	NA	NA	NA
	Overwinter	M	6.79 (1,19)	0.012	0.35 (1,19)	0.560	NA	NA
		F	4.16 (1,19)	0.06	8.41 (1,19)	0.006	NA	NA
								
%Water	S. Ontario to Texas	M	58.62 (4,249)	<0.001	14.0 (4,199)	<0.001	48.17 (4,160)	<0.001
		F	17.54 (4,195)	<0.001	15.59 (4,127)	<0.001	61.58 (4,102)	<0.001
	N to central Mexico	M	51.59 (2,252)	<0.001	NA	NA	NA	NA
		F	46.9 (2,200)	<0.001	NA	NA	NA	NA
	Overwinter	M	6.69 (1,19)	0.02	1.6 (1,19)	0.212	NA	NA
		F	0.22 (1,19)	0.646	2.52 (1,19)	0.121	NA	NA
								
Wing loading	S. Ontario to Texas	M	11.52 (4,249)	<0.001	1.2 (4,199)	0.331	28.06 (4,160)	<0.001
		F	10.92 (4,195)	<0.001	3.78(4,127)	0.006	5.97 (4,102)	<0.001
	N to central Mexico	M	18.8 (2,252)	<0.001	NA	NA	NA	NA
		F	7.4 (2,200)	<0.001	NA	NA	NA	NA
	Overwinter	M	1.67 (1,19)	0.213	0.746 (1,19)	0.393	NA	NA
		F	12.97 (1,19)	0.002	0.306 (1,19)	0.583	NA	NA

In 2019, a striking decline in lipid reserves was observed in Texas and northern Mexico compared with the more northern sites but increased again in central Mexico and at the two overwintering sites (Table 1, Fig 2; males: *F*_1,82_ = 13.71, *P* < 0.001; females: *F*_1,72_ = 47.83, *P* < 0.001). There were no changes in percent water (Table 1, Fig 3; males: *F*_1,82_ = 0.59, *P* = 0.441; females: *F*_1,72_ = 0.202, *P* = 0.654) between the three regions and while wing loading for males did not change (Table 1, Fig 4; *F*_1,82_ = 1.61, *P* = 0.208) it increased for females (*F*_1,72_ = 10.62, *P* = 0.002).

In 2020, lipid levels were higher overall than the previous year and, with the exception of Oklahoma where few monarchs were observed nectaring, there were no noticeable declines through the migratory transect. Male monarchs showed no change in lipid levels between the three regions (Table 1; *F*_1,89_ = 1.02, *P* = 0.315) but did increase in percent water (*F*_1,89_ = 39.23, *P* < 0.001) and wing loading (*F*_1,89_ = 49.08, *P* < 0.001) close to the overwintering site following a decline in Texas, while for females, all of these parameters increased (lipids: *F*_1,75_ = 18.11, *P* < 0.001; percent water: *F*_1,75_ = 40.57, *P* < 0.001; wing loading: *F*_1,75_ = 55.64, *P* < 0.001) between northern Mexico and the overwintering sites. As noted earlier, the monarchs in Iowa were either nectaring or roosting at the time of collection in 2019 and 2020. Interestingly, in 2019, roosting monarchs had greater proportions of lipid vs. dry mass than nectaring monarchs (roosting: 87.9 ± 43.5%, n = 109; nectaring: 20.4 ± 12.5%, n = 66; t = 151.6, *P* < 0.001), while the inverse was observed in 2020 (roosting: 37.7 ± 27.9%, n = 14; nectaring: 50.5 ± 19.8%, n = 60; t = 4.0, *P* = 0.05).

The 2021 lipid levels tended to be lower than in 2020 but, with the exception of Kansas and Oklahoma, were overall higher than in 2019 (Fig 2, Supplementary Table S1). Again, between Texas and northern Mexico, and the overwintering sites monarchs increased in body lipids (males; *F*_1,116_ = 15.21, *P* < 0.001; females: *F*_1,103_ = 25.06, *P* < 0.001; Fig 2) and wing loading (males: *F*_1,116_ = 57.77, *P* < 0.001; females: *F*_1,103_ = 35.72, *P* < 0.001; Fig 4) but did not show changes in percent water (males: *F*_1,116_ = 0.03, *P* = 0.87; females: *F*_1,103_ = 0.05, *P* = 0.83; Fig 3).

As we had larger sample sizes in 2019, we examined morphological aspects of migrant monarchs. As expected, forewing length (male: 5.17 ± 0.24 cm; female 5.15 ± 0.26 cm) was correlated with total wing area for both male (r^2^ = 0.62, *P* < 0.001) and female (r^2^ = 0.63, *P* < 0.001) monarchs. Males had larger wing surface area (GLM, *F*_1,948_ = 5.275, *P* = 0.022) which also differed among sites (*F*_13,948_ = 3.063, *P* < 0.001) but with no interaction between sex and site (*F*_13,948_ = 0.086, *P* = 0.882).

To facilitate the interpretation of the NDVI results both geographically and temporally, the collection sites were sorted into three groups (Supplementary Fig S2). Group A consists of the four locations north of Texas. The NDVI Z scores were positive through the migrations but consistently negative for Ames in 2019. However, that was not reflected in the lipid values which were high. Group B consisted of Comfort, Texas, and the two sites in northeast Mexico. The negative NDVI scores for Comfort and Ciudad Victoria for 2019 show the influence of the drought while the value for Monterrey, the westernmost location, was positive. Curiously, negative values for this location persisted through 2021 and was the only Mexican location to have negative values in 2021. In group C, the negative NDVI scores for 2019 extended as far as San Luis Potosi showing the southern extent of the drought. In contrast, values were positive for all other highland locations in Mexico. The values for all highland areas were negative in 2020 suggesting drought conditions (Supplementary Table S3). The only overwintering samples for that year were the 10 of each sex collected at the overwintering sites. Wet weights and lipids of this sample were in the normal range, but the samples may have been too few to show an effect of a drought.

## Discussion

Beall (1948) suggested that monarchs accumulate lipids at the start of migration to provide the resources required to support the rest of the journey, including the overwintering period, while more recent investigations have suggested an increase in lipid occurs towards the end migration, closer to the overwintering sites (Brower, 1985; Masters *et al.,* 1988; Brower *et al.,* 2015; Hobson *et al.,* 2020). While migrating during the fall, eastern North American monarchs clearly nectar along the route at the many “stopover” sites and our results underline the marked within- and between-year differences that can occur at different sites along the migratory pathway. However, the ability to increase their lipid loads for overwintering while close to the Mexican overwintering sites has a number of important ramifications related to their ability to withstand adverse conditions along the route. Presumably, the ability to compensate for low lipid acquisition due to poor nectaring conditions north of wintering sites will increase the probability reaching and surviving at the overwinter sites (Taylor *et al.,* 2020).The fall monarch migration is especially complex and, as a result, there are several (not mutually exclusive) reasons for differences we observed in the various parameters measured. For example, the timing and pace of migrations is linked to the declining angle of the sun at solar noon (SASN); Taylor *et al.* (2019) reported that 90% of the tagged monarchs recovered in Mexico were tagged when the SASN values were between 57° and 46°. Sun angle at our sites during the 3 years of collection (Supplementary Table S1) ranged from 43.5° (Lawrence, Kansas 9–14 October 2020) to 55.4° (London, Ontario 6 Sept 2021). Depending on the site, our sampling dates could be considered as early or late with respect to this ±30 day “migration window.” The Stillwater, Oklahoma, monarchs were collected late in the window but coincided with their presence in the area, and in all 3 years these individuals had the lowest lipid levels observed north of Mexico. However, to what extent these levels were the result of low nectar availability due to the timing of arrival is not clear. Furthermore, the size of the butterflies and sex ratio changes during the migratory period, with the leading edge being dominated by large males while trailing migrants tend to be smaller with a higher proportion of females (Becker, 2008; Taylor, unpublished data). Similarly, what the insects were doing at the time they were collected could have an influence. At the majority of sites in this study, the collections consisted of nectaring individuals during the day, but in 2019 and 2020 the Iowa samples included individuals collected during the day while others were obtained at nocturnal roost sites. A comparison of the two groups showed significant differences in lipid content but differed dramatically in the direction of the effect between years. Although it was not our objective to determine proximate or ultimate causal factors driving lipid levels in migrating monarchs, weather, as reflected by temperatures, precipitation, strong headwinds and storms, both before and during the migrations, clearly contributed to the variability we found. Reproduction or migrations delayed by high temperatures are associated with lower numbers of hectares with wintering monarchs in Mexico (Taylor *et al.,* 2019, 2020).

The maximum temperature attained each day and the daytime conditions during which the migration occurred during the migration windows for each location and year are summarized in Supplementary Table S3. While the optimal conditions for migratory flight have not been defined, it is apparent that there is little to no migratory flight when temperatures are below 13°C and relatively little when temperatures exceed 29°C. The optima for linear migratory flight are probably between 19°C and 24°C. Temperatures above 30°C, associated with slower migrations as seen in 2019 and 2021, occurred at more southerly latitudes in the United States (Fig S1) although daily high temperatures generally ranged from 24°C to 29°C at 1800 m in the Mexican highlands.

Variation in reported monarch lipid levels also results from the relatively short periods of sampling we conducted at each site and the variation that can be expected in the phase of the migration or even time of day. Gibo and McCurdy (1993) investigated lipid levels in migrating monarchs in southern Ontario and found that these levels differed considerably from the early to middle to late phase of migration reflecting the time available for nectar foraging and the availability of nectar. These results point to the need to account for timing of sampling, the abundance of floral resources and weather along the migratory route in order to interpret lipid levels and where those lipids used for overwintering are acquired (Hobson *et al.,* 2020; Pilecky *et al.,* 2023).

Compared with the other 2 years of our study, the 2019 drought in Texas appeared to have a negative impact on lipid acquisition and retention (Supplementary Table S2, Fig. 2). This drought was not unique as a similar situation was reported by Brower *et al.* (2015). A scan through the Palmer Drought Monitor postings for mid-October indicates that droughts in Texas of approximately the same or greater severity occurred during 12 of the 23 years in that record. Thus, fall droughts are relatively common in Texas and are a hazard for monarchs since droughts are associated with lower-than-expected numbers of monarchs reaching overwintering sites (Saunders *et al.,* 2019). While this interpretation points to the importance of maintaining and creating nectar sources in Texas, it also points to the importance of the floral resources in the Mexican highlands since the less drought prone conditions there presumably constitute a more reliable source of nectar for monarchs as they approach the overwintering areas. Unfortunately, looking ahead, the frequency and severity of droughts in Texas is expected to increase in the coming decades (Cook *et al.,* 2019; Nielsen-Gammon *et al.,* 2021).

The lack of rainfall in the months preceding the migration in 2019 resulted in a moderate to extreme drought in Texas yet the mean temperatures in October 2019 were almost identical to those in 2021 when there was no drought and lipid levels were higher (Supplementary Table S3). These data support the interpretation that the low lipids in 2019 were the result of a lack of nectar. The impact of drought conditions will have a direct effect on the foraging of adults (Frey *et al.,* 2002; Forrest, 2015) and the availability of flower density. However, even if there are flowers present, the accessibility of the nectar will be affected by the weather, although the degree to which this occurs will be dependent on flower morphology (Nicolson, 1998, 2022). Under hot, dry conditions the viscosity of nectar will increase due to water loss and such changes can affect both the concentration of nectar and the energy obtained during a feeding bout (Pivnick and McNeil, 1985). Similarly, such conditions may change the quality and composition of the nectar produced (Descamps *et al.,* 2021). Furthermore, at any given time, nectar availability will be affected by the level of competition with other nectivorous species, such as bees who, due to their more flexible means of obtaining nectar (Wei *et al.,* 2020), may be better at exploiting the available nectar sources.

The idea that the low level of lipids observed in Texas in 2019 was driven by the drought is supported by the low NDVI measure of greenness for 2019 relative to the higher measures in 2020 and 2021 (Supplementary Table S3, Supplementary Fig S2). In this case, NDVI measures serve as a surrogate for nectar availability (Saunders *et al.,* 2019). The use of NDVI to approximate nectar availability by recording deviations from long-term averages is a more fine-grained approach to measuring vegetative productivity and has a number of advantages. First, areas with no or little nectar, such as water, forests, and cropland, are excluded so that only potential nectar production regions are measured. Second, it is an index that can be applied to areas of different sizes and time intervals. Third, this approach, if applied over a decade or more provides an historical record of average conditions. However, it remains unclear just how well NDVI values can be used in future investigations because greenness is not necessarily an indication of floral phenology or nectar production per se. Nonetheless, we encourage future research examining how well NDVI can be used to examine the physiology of fueling in migratory insects and other animals.

While it seems logical to link late fall lipid levels with overwintering success and recruitment into the subsequent breeding population, data on fall lipid levels are needed for a series of years to determine whether such relationships exist. Such assessments would require accurate measures of overwintering mortality, as well as the mortality as the monarchs return to Texas from the overwintering sites in March, a journey of at least 1280 km. The cause-and-effect relationships in this case are also confounded by the fact there are several stages, involving three to five generations, between one overwintering population and the next. Still, further study is needed of the acquisition of lipids as monarchs pass through the Mexican highlands in the fall and the utilization of stored lipids during the winter. These in turn should be followed by studies of how lipids are depleted and restored as monarchs transition from a non-reproductive condition to a migratory and reproductive state in March. Of course, a worst-case scenario would be drought conditions extending throughout the nectar corridor from the southern US to the Mexican wintering roost sites, especially if then followed by challenging overwintering weather conditions.

Although documenting lipid levels in migrating monarchs was our primary interest, we opportunistically measured water content and wing loading. Gibo (1993) suggested that water as ballast plays an important role in wing loading thus linking flight dynamics with water content and lipid mass to glide ratio and flight speed. Our data show lower differences in water content than in lipids, both within and between years at all sites, indicating that while nectar may be a source of water, monarchs are using other sources, such as dew or free water (Frey *et al.,* 2002).

As several studies have shown, migrants tend to have larger wings than non-migrants (Becker, 2008; Altizer *et al.,* 2015; Flockhart *et al.,* 2017) as this favors lift, which combined with high wing loading, affect both flight speed and gliding (Gibo, 1993; Becker, 2008; Altizer and Davis, 2010; Li *et al.,* 2016). Becker (2008) found that large males predominate in collections made of monarchs on the leading edge of a migration which is consistent with the expectation that larger size, within limits, favors faster flight. Large size also helps to counter opposing winds (Piggott, 1976; Lighthill, 1977; Welch *et al.,* 1977). In this study, the lowest wing loading occurred during the 2019 migration, which was both the latest migration and the one with the most negative NDVI scores (Supplementary Table S3). Both are indications of low nectar availability. With the exception of the northern Mexico (Monterrey, Nuevo Leon) location, the next 2 years showed fairly constant wing loading through migration followed by highest levels at the wintering sites, as expected.

Our results emphasize the importance of suitable nectar foraging sites, especially through the Mexican portion of the migration (Hobson *et al.,* 2020) and underlines not only both the hazards posed by conditions limiting nectar availability but also the resilience of this species. Past research into continental populations of the eastern migratory monarch population has focused predominantly on factors operating on the breeding and wintering grounds (but see Brower *et al.,* 2015, 2017; Hunt and Tongen, 2017). Few studies have considered eco-physiological connections linking weather and population status (Wilcox *et al.,* 2019), and there is a paucity of studies on direct effects of weather on any stage during the lifecycle (Couture *et al.,* 2015; Ethier and Mitchell, 2023). Furthermore, our findings emphasize the critical need to fill these gaps during the migratory phases. We recognize that an important limitation of our study is that at any given sampling location, we only sampled survivors and could not account for individuals that suffered mortality between sites. However, ours was a comparative study of fuel loads at the population and not individual level and it is currently not clear how feasible it is to track individual survivorship with individual lipid levels throughout migration.

Our findings also suggest possible avenues for conservation. There has been considerable interest in the conservation of migratory monarch butterflies in North America (Flockhart *et al.,* 2015; Oberhauser *et al.,* 2017; Thogmartin *et al.,* 2017a, 2017b; Malcolm, 2018; Wilcox *et al.,* 2019; Taylor *et al.,* 2020; Zylstra *et al.,* 2021) with most considering the breeding (Lark *et al.,* 2015; Pleasants, 2017) and wintering grounds (Williams and Brower, 2015; Oberhauser *et al.,* 2017; Saunders *et al.,* 2019; Zylstra *et al.,* 2021). In contrast while the importance of nectar has been recognized (Inamine *et al.,* 2016; Agrawal and Inamine, 2018), until now, there has been very little attention giving possible interventions during the migratory phase. From this perspective, it is essential that a better understanding of the preferred species of nectar sources used by monarchs at different sites along the migratory pathway is established and that these resources are readily available, particularly in the regions close to the overwintering sites.

## Supplementary Material

Web_Material_coad087

## Data Availability

The data underlying this article will be shared on reasonable request to the corresponding author.
