# Supplementary material for: Dynamics of stored lipids in fall migratory monarch butterflies (Danaus plexippus): Nectaring in northern Mexico allows recovery from droughts at higher latitudes
Source: Conserv Physiol. 2023 Nov 24;11(1):coad087. doi: 10.1093/conphys/coad087 (PMC10673816; doi:10.1093/conphys/coad087)
Supplement: Web_Material_coad087 [file web_material_coad087.pdf]

SUPPLEMENTARY MATERIAL

Dynamics of stored lipids in fall migratory Monarch Butterflies (*Danaus plexippus*): Nectaring in northern Mexico allows recovery from droughts at higher latitudes

Keith A. Hobson[1,2*], Orley Taylor[3], M. Isabel Ramírez[4], Rogelio Carrera-Treviño[5], John Pleasants[6], Royce Bitzer[7], Kristen A. Baum[8], Blanca X. Mora Alvarez[1], Jude Kastens[9] and Jeremy N. McNeil[1]

[1]Department of Biology, University of Western Ontario, London, ON, Canada.

[2] Environment and Climate Change Canada, Saskatoon, SK, Canada

[3]Department of Ecology and Evolutionary Biology and Kansas Biological Survey & Center for Ecological Research, University of Kansas, Lawrence, Kansas, USA

[4]Centro de Investigaciones en Geografia Ambiental, Universidad Nacional Autónoma de Mexico, Morelia, Michoacán, Mexico

[5]Facultad de Medicina Veterinaria y Zootecnia, Universidad Autónoma de Nuevo León, Escobedo, Nuevo León, México

[6]Department of Ecology, Evolution, and Organismal Biology, Iowa State University, Ames, Iowa, USA

[7]Department of Plant Pathology, Entomology, and Microbiology, Iowa State University, Ames, Iowa

[8]Department of Integrative Biology, Oklahoma State University, Stillwater, Oklahoma, USA

[9]Kansas Biological Survey & Center for Ecological Research, University of Kansas, Lawrence, Kansas, USA

Table S1. Summary data for sites used to sample fall monarch butterflies in 2019, 2020, 2021. Site numbers correspond to those in Table S2. Elevation values were obtained from https://www.freemaptools.com/elevation-finder.htm. SASN represents the sun angle at solar noon, a marker for the progress of the migration and the phenology of fall flowering. Ninety percent of the monarchs recovered in Mexico were tagged when the SASN was between 57-46 degrees. Monarchs tagged when SASN was below 46 degrees are seldom recovered in Mexico (Taylor, et al., 2019). Elevation groups are described in the caption of Table S4.

Region (Site)	Year	Date	Lat	Lon	Elevation (m)	Male	Female	SASN
Migration:					Group A			
London, ONT (1)	2019	30 Aug-6 Sept	42.9581	-81.2933	268	19	13	54.1
		17 Sept	43.8118	-81.7216	204	13	22	49.2
		27 Sept	42.6150	-80.4588	179	15	12	46.1
	2020	1 Sept	43.7341	-81.7165	213	18	13	55.2
		3 Sept	42.62357	-80.45014	186	13	10	54.5
		3 Sept	42.66921	-81.16989	209	12	8	54.5
		3 Sept	42.96077	-81.29900	241	9	6	54.5
	2021	2-10 Sept	42.66921	-81.16989	209	22	17	53.4
		6 Sept	43.7341	-81.7165	213	6	6	55.4
Ames, IA (2)	2019	11 Sept	42.07192	-93.6383	287	36	15	52.6
		20 Sept	42.07192	-93.6383	287	13	19	49.9
		25-26 Sept	42.01439	-93.6523	298	25	15	46.8
		30 Sept	42.0046	-93.6356	285	7	5	45.1
		8-9 Oct.	42.00366	-93.6579	302	16	26	44.9
	2020	7 Sept	42.07192	-93.63827	287	10	4	53.9
		14-22 Sept	41.98816	-93.65778	308	34	27	49.7
	2021	20 Sept	41.98816	-93.65778	308	11	11	49.0
Lawrence, KS (3)	2019	27 Sept-14 Oct	38.91573	-95.23330	251	36	28	49.5-45.2
	2020	16-25 Sept	38.91573	-95.23330	251	42	26	51.6
		9-14 Oct	38.91573	-95.23330	251	3	3	43.5
	2021	21 Sept-10 Oct	38.91573	-95.23330	251	84	34	51.3-44.1
Stillwater, OK (4)	2019	5 Oct	36.12760	-97.07372	286	15	15	48.9
		8 Oct	36.12760	-97.07372	286	23	7	47.7
		12-15 Oct	36.12760	-97.07372	286	14	9	45.8
	2020	10 Oct	36.12760	-97.07372	286	21	14	47.1
	2021	2-16 Oct	36.12760	-97.07372	286	51	28	50.2-44.9
					Group B			
Comfort, TX (5)	2019	14 Oct	30.29913	-98.79564	500	3	1	51.3
		15 Oct	29.40480	-99.29157	304	13	7	51.4
		23 Oct	29.40480	-99.29157	304	6	4	48.6
	2020	10 Oct	29.97279	-98.83673	413	3		53.3
		22 Oct	29.94531	-98.90078	464	1	1	48.9
		11 Oct	29.79532	-99.23947	431	11	6	52.9
		13-20 Oct	29.89214	-98.67181	359	13	8	50.7
	2021	14-30 Oct	29.97279	-98.83673	413	19	11	52.5-44.3
Monterrey, MX (6)	2019	21 Oct	25.67165	-100.49646	890	41	41	53.4
	2020	22 Oct	25.67165	-100.49646	890	44	44	53.2
	2021	23-25 Oct	25.67165	-100.49646	890	39	33	52.5
Ciudad Victoria, MX (7)	2019	4 Nov	23.63472	-99.19556	858	25	28	50.7

Table S1 (cont.)

					Group C			
San Luis Potosí, MX (7)	2019	1-2 Nov	22.21584	-100.61195	1819	49	31	52.9
Queretaro, MX (8)	2019	7 Nov	20.72072	-100.44305	1967	56	25	52.8
Guanajuato, MX (8)	2019	8 Nov	20.14182	-100.69838	1957	40	40	53.2
Contepec, MX (8)	2019	6 Nov	19.87598	-100.16697	2270	18	22	53.2
Senguio, MX (8)	2019	6 Nov	19.71300	-100.33800	2460	25	15	53.8
**Overwinter:**								
Sierra Chincua, Angangueo, MX (9)	2019	27 Dec	19.67203	-100.29489	3330	10	10	NA
	2020	25 Nov-2 Dec	19.67203	-100.29489	3330	20	20	NA
	2021	17 Nov-22 Dec	19.67203	-100.29489	3330	60	60	NA
Cerro Pelon, Macheros, MX (10)	2019	26 Dec	19.38825	-100.26098	3098	10	10	NA
	2020	26 Nov-10 Dec	19.38525	-100.26098	3098	20	20	NA

Table S2. Summary data (mean ± SD) of morphometrics and body condition parameters measured for monarch butterflies sampled 2019 to 2021 at sites indicated in Figure 1. Groups are those sites common to all years and used to investigate inter-year patterns shown in Figures 2-4. The percent lean mass was calculated for the lipid extracted body only (i.e. minus wings).

Location (site, group)	Sex	Year	N	Wing area (cm^2)	Wet wt (g)	Dry wt (g)	% Water	Lipid (mg)	% of wet wt	% of dry wt	% lean mass	Wing loading (mg/cm^2)
Ontario (1,1)	M	2019	47	35.37±2.60	0.534±0.070	0.212±0.062	59.9±8.3	63.7±37.0	11.4±5.6	27.7±9.7	47.2±26.1	15.2±1.6
	F	2019	47	35.05±3.02	0.492±0.079	0.197±0.067	60.7±7.8	57.0±36.6	11.1±5.8	27.2±11.2	45.8±28.1	14.0±1.6
	M	2020	69	35.09±2.06	0.518±0.076	0.242±0.047	53.4±5.0	54.9±34.2	10.1±5.3	21.1±9.7	39.3±23.2	14.8±1.8
	F	2020	46	34.5±3.10	0.478±0.082	0.230±0.048	51.9±4.8	54.9±36.9	11.0±6.2	22.2±10.9	43.1±28.4	13.8±1.8
	M	2021	26	35.7±2.61	0.558±0.065	0.248±0.042	55.8±6.5	44.6±30.6	7.7±5.0	17.0±10.9	20.5±12.1	15.6±1.2
	F	2021	23	36.4±2.38	0.513±0.064	0.221±0.039	57.2±2.6	52.7±45.7	9.8±7.4	22.5±16.2	21.5±19.2	14.1±1.6
Iowa (2,2)	M	2019	96	34.02±2.87	0.514±0.110	0.271±0.089	48.3±8.3	82.7±66.1	14.5±9.4	26.9±15.6	62.6±46.8	15.0±2.6
	F	2019	80	33.74±3.06	0.471±0.112	0.251±0.091	47.9±8.4	76.5±65.7	14.3±9.8	25.9±15.9	63.0±50	13.9±2.4
	M	2020	43	34.63±2.71	0.490±0.065	0.242±0.043	49.3±5.0	61.4±31.6	12.2±5.2	24.2±9.2	45.7±22.4	14.1±1.6
	F	2020	30	34.08±2.8	0.463±0.052	0.243±0.034	52.4±4.7	65.3±25.1	13.9±4.9	26.2±7.8	51.5±21.0	13.6±1.5
	M	2021	11	32.24±2.78	0.441±0.060	0.22±0.064	49.7±4.5	61.0±31.0	13.3±5.9	26.1±10.4	32.6±12.7	13.7±1.9
	F	2021	11	34.80±2.78	0.539±0.085	0.321±0.064	40.7±3.4	126.9±35.8	23.3±3.4	39.3±4.3	51.0±5.2	15.4±1.7
Kansas (3,3)	M	2019	36	33.66±3.10	0.500±0.079	0.213±0.047	57.4±4.4	39.3±33.3	7.5±5.9	16.5±12.1	30.0±26.0	14.8±2.0
	F	2019	29	33.73±2.58	0.463±0.132	0.190±0.055	58.6±4.0	26.9±33.8	5.0±5.6	11.4±11.5	20.8±24.8	13.6±3.2
	M	2020	43	35.02±2.61	0.494±0.062	0.251±0.045	49.5±4.4	58.6±33.5	11.6±6.3	22.4±10.8	41.5±24.6	14.1±1.5
	F	2020	27	34.58±2.62	0.450±0.074	0.229±0.045	49.1±5.1	48.0±48.6	10.0±9.4	19.4±17.0	37.4±39.4	13.0±1.9
	M	2021	55	34.90±2.87	0.509±0.078	0.202±0.033	60.4±2.1	26.9±15.1	5.2±2.6	12.9±6.0	15.9±7.1	14.6±1.5
	F	2021	33	32.9±3.14	0.46±0.07	0.185±0.030	59.6±3.3	26.9±22.6	5.7±4.6	13.7±9.3	16.5±11.2	13.5±2.8
Oklahoma (4,4)	M	2019	52	34.93±3.37	0.460±0.068	0.176±0.028	61.8±2.5	8.1±11.4	1.7±2.4	4.4±5.5	6.8±10.9	13.2±1.3
	F	2019	31	33.84±2.80	0.393±0.062	0.158±0.021	59.4±4.1	6.0±5.8	1.5±1.4	3.7±3.4	5.6±5.5	11.6±1.2
	M	2020	21	35.5±2.40	0.511±0.066	0.222±0.038	56.7±3.7	28.2±29.0	5.3±5.1	11.5±10.1	14.4±12.3	14.4±1.7
	F	2020	14	34.2±4.35	0.448±0.079	0.196±0.045	56.6±3.3	21.0±24.5	4.4±4.4	9.6±8.7	12.4±11.0	13.0±1.1
	M	2021	53	33.76±3.76	0.442±0.076	0.183±0.045	58.9±3.6	13.7±25.3	2.7±3.9	6.0±7.7	7.9±9.4	13.1±1.4
	F	2021	28	33.0±3.80	0.417±0.083	0.178±0.051	57.6±4.3	14.2±36.1	2.9±5.6	6.0±9.3	7.8±11.8	12.6±1.9
Texas (5,5)	M	2019	22	35.70±2.46	0.574±0.048	0.197±0.033	65.8±3.6	19.0±19.5	3.2±3.1	8.8±7.3	13.9±13.0	16.1±1.2
	F	2019	12	32.27±2.56	0.563±0.047	0.201±0.041	64.5±4.8	31.9±31.3	5.4±4.9	13.9±11.1	25.6±24.2	16.0±1.2
	M	2020	27	34.54±2.51	0.503±0.082	0.247±0.054	51.2±4.0	69.7±40.8	13.2±6.4	26.3±10.9	52.8±28.9	14.5±1.9
	F	2020	14	34.35±2.55	0.495±0.071	0.260±0.050	47.6±5.0	88.8±45.1	17.6±7.9	32.6±12.6	75.5±40.2	14.4±1.7

	M	2021	19	34.12±2.74	0.398±0.077	0.197±0.045	50.6±5.6	29.5±25.9	7.0±5.4	13.6±8.9	39.7±26.8	11.6±1.9
	F	2021	11	33.48±4.10	0.392±0.095	0.213±0.061	45.8±6.8	49.6±39.7	11.8±7.2	20.9±11.1	22.8±18.6	11.6±1.6
Monterrey (6,6)	M	2019	41	33.80±2.67	0.435±0.070	0.214±0.051	51.1±5.1	36.7±38.9	7.9±6.6	15.2±11.3	29.7±31.3	12.9±1.7
	F	2019	41	33.24±2.88	0.374±0.069	0.197±0.046	47.4±4.6	29.8±27.1	7.5±5.7	13.7±9.8	24.3±20.3	11.2±1.5
	M	2020	43	35.37±2.85	0.384±0.079	0.253±0.047	32.9+11.2	71.9±2.8	18.9±6.4	27.9±8.1	65.0±50.1	10.8±1.8
	F	2020	42	34.43±2.90	0.334±0.085	0.239±0.057	27.5±8.0	68.5±33.4	20.2±6.8	27.5±8.5	64.4±29.7	9.6±2.3
	M	2021	39	35.09±3.50	0.282±0.067	0.225±0.047	19.4±8.1	71.1±40.5	24.8±9.8	30.8±11.7	44.8±33.4	8.12±2.2
	F	2021	33	34.87±3.05	0.268±0.059	0.217±0.043	17.9±11.1	75.3±36.8	28.2±13.2	34.3±15.6	46.0±24.6	7.80±2.0
Ciudad Victoria (7,7)	M	2019	25	33.31±2.09	0.376±0.060	0.207±0.041	44.9±6.2	31.4±28.0	7.9±5.9	13.9±9.5	24.6±21.6	11.3±1.7
	F	2019	28	33.56±3.06	0.339±0.046	0.195±0.029	42.2±6.4	28.7±21.0	8.3±5.4	14.0±8.1	25.4±18.8	10.1±1.0
San Luis Potosí (8,7)	M	2019	49	33.90±2.21	0.583±0.075	0.315±0.056	46.2±5.1	120±45.9	18.3±6.2	36.9±9.6	82.3±30.3	17.2±2.1
	F	2019	31	34.39±2.27	0.560±0.095	0.321±0.074	43.2±5.6	132±60.0	22.7±8.0	39.1±11.7	98.0±41.9	16.3±2.5
Querétero (9,8)	M	2019	56	34.83±2.47	0.442±0.073	0.251±0.044	42.9±5.1	61.8±28.7	13.7±5.1	23.8±7.7	45.8±20.5	12.6±1.7
	F	2019	25	34.43±2.43	0.430±0.076	0.260±0.050	39.7±4.4	76.9±34.9	17.3±6.1	28.5±9.3	63.5±29.1	12.5±1.7
Guanajuato (10,8)	M	2019	40	34.48±2.78	0.532±0.086	0.295±0.061	44.7±4.1	85.8±46.7	18.3±6.2	32.7±9.4	75.4±51.0	15.4±2.0
	F	2019	40	33.83±2.08	0.518±0.084	0.308±0.056	40.4±5.8	122.8±39.0	23.6±5.9	39.1±7.0	99.2±37.8	15.3±2.1
Contepec(11,8)	M	2019	18	34.38±1.78	0.404±0.050	0.263±0.037	34.8±5.2	80.4±28.3	19.7±6.1	29.8±7.5	62.1±22.2	11.7±1.2
	F	2019	22	34.48±1.75	0.385±0.061	0.272±0.053	29.8±5.0	89.9±42.0	22.3±8.8	31.2±11.2	75.6±36.6	11.2±1.7
Senguio(12,8)	M	2019	25	34.95±2.71	0.385±0.076	0.242±0.049	36.8±5.5	56.8±37.1	14.1±7.7	21.8±10.8	42.0±26.3	11.0±1.7
	F	2019	15	33.8±1.16	0.338±0.055	0.228±0.037	32.1±5.8	61.7±27.0	17.9±6.3	26.1±8.4	54.9±24.7	10.0±1.7
Sierra Chincua(13,9)	M	2019	10	35.74±2.60	0.547±0.079	0.258±0.040	52.8±2.5	78.4±28.4	14.1±4.0	29.7±7.2	60.2±21.0	15.3±1.6
	F	2019	10	34.0±2.88	0.521±0.073	0.253±0.042	51.6±3.4	92.3±27.9	17.6±4.0	36.0±6.4	84.4±24.3	15.3±1.1
	M	2020	10	35.2±3.3	0.599±0.157	0.240±0.046	58.0±10.2	75.3±28.5	13.2±5.4	30.5±7.6	65.3±23.9	17.1±4.5
	F	2020	10	34.7±3.3	0.571±0.152	0.241±0.054	54.8±15.3	85.8±39.4	16.3±8.7	33.8±9.9	81.9±33.5	16.6±4.4
	M	2021	59	34.5±3.4	0.610±0.118	0.301±0.070	50.2±9.1	127±48.9	20.5±6.4	41.3±11.3	55.2±15.4	17.7±3.4
	F	2021	60	34.5±2.8	0.630±0.132	0.330±0.081	46.5±10.9	156.9±59.7	24.9±7.5	46.7±11.8	65.1±17.1	18.2±3.6
Cerro Pelón(14,10)	M	2019	10	35.48±3.07	0.504±0.062	0.219±0.051	56.6±4.0	47.3±25.5	9.0±3.6	20.5±6.7	37.9±17.0	14.1±2.3
	F	2019	10	34.51±2.08	0.455±0.061	0.217±0.059	52.8±7.8	60.8±45.1	12.6±8.1	25.1±12.6	55.8±37.1	13.2±1.5
	M	2020	10	35.65±2.08	0.569±0.110	0.255±0.045	53.0±14.2	85.4±35.7	15.9±8.1	32.2±9.2	70.3±29.0	16.0±2.3
	F	2020	10	35.59±3.56	0.559±0.128	0.287±0.071	46.6±17.1	125.1±49.1	23.0±10.1	42.2±7.9	113.3±35.1	15.8±3.8

Table S3. Mean maximum temperatures (ºC) and deviations from long-term average mean maximum for the months and locations indicated. The months represent the main migration intervals for each location. The deviations were generally above those of the long-term means for 2019 and 2021 and more similar to the long-term means for 2020. Temperatures for Comfort, Texas were similar for all three years.

Location	Month	2019	2020	2021	Long-term mean
London, ON	Sep	22.6 (+3.8)	20.9 (+2.1)	21.7 (+2.9)	18.8
Ames, IA	Sep	27.3 (+2.4)	23.7 (-1.2)	27.9 (+3.0)	24.9
Lawrence, KS	Sep	30.3 (+3.1)	26.3 (-0.9)	30.1 (+2.9)	27.2
Stillwater, OK	Oct	20.7 (-1.9)	21.4 (-1.2)	24.8 (+2.2)	22.6
Comfort, TX	Oct	28.6 (+0.4)	29.2 (+1.0)	29.0 (+0.8)	28.2

Figure S1. Percentage of days (y axis) with indicated temperatures (ºC) during the migrations for the three years of this study on the x-axis. The intervals include the 15 days before SASN dropped below 57 degrees for each location since the conditions during those intervals influence the timing of the migration reaching each location. Mean maximum temperatures increased with decreasing latitudes. These temperatures were highest in 2021 but were similar in 2019. The temperatures in 2020 were lower and the pace of the migration was similar to the 1990s as reflected in the collection dates (Table S1).

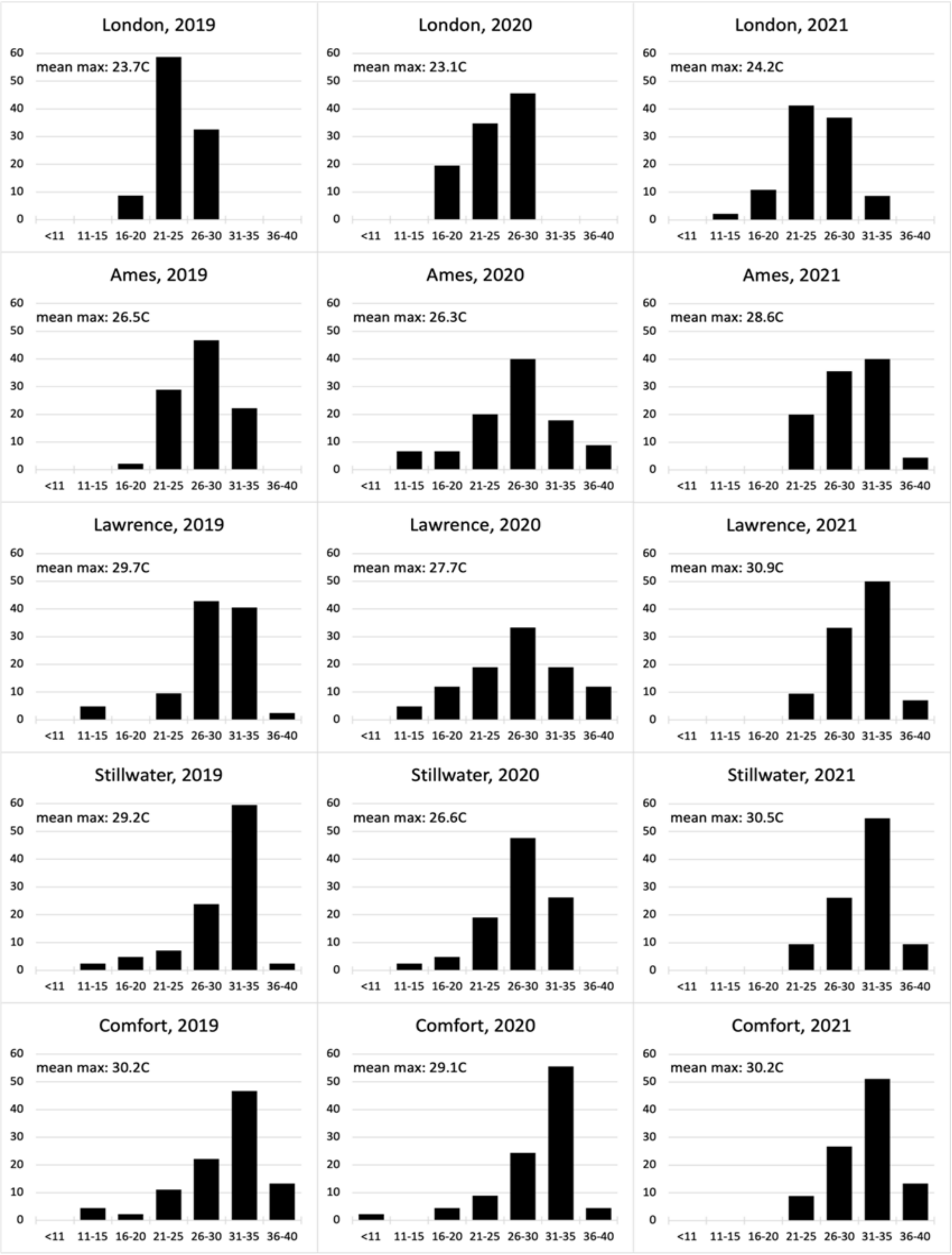

NDVI Calculations and interpretation

Weekly NDVI time series data preparation methods were adapted from Brown *et al.* (2013) and Wardlow *et al.* (2006). The NDVI signal used to represent each sample site is an average across NDVI signals from nearby pixels (within 50 km) primarily covered by grassland or shrubland according to the ESA WorldCover 2021 dataset distributed through https://esa-worldcover.org/en (Figure S2). To get to a singular annual NDVI-based variable reflective of vegetative vigor of plants producing nectar in the vicinity of the sample sites around the arrival time of the fall migration, NDVI values from a manually selected, site-specific, 8-week interval were summed. At each site, the sample mean and standard deviation were then computed from the 11 years of observations of the NDVI sum, and these statistics were used to transform the NDVI sum values to Z-scores to simplify interpretation.

NDVI Data Preparation

Source data for the Normalized Difference Vegetation Index (NDVI) assessment are multi-day composite VIIRS (Visible Infrared Imaging Radiometer Suite) NDVI time series prepared using the conterminous U.S. (CONUS) and Central America-Mexico (CAM) collections developed by USGS and available for download through the USGS Download Data Service (https://dds.cr.usgs.gov/). Temporal coverage is 2012-2022 and pixel size is approximately 375m. The CONUS collection consists of 7-day composite NDVI scenes issued every 7 days, implying no overlap between consecutive composite periods. The CAM collection consists of 10-day composite NDVI scenes issued every 5 days, implying five days of overlap between consecutive composite periods. Subsets from both time series raster datasets were extracted using a mask comprised by 50-km circular buffers around each monarch sampling location.

Each multi-day composite VIIRS NDVI scene is accompanied by a date-of-acquisition (DOA) layer containing values that indicate from which calendar day in the composite period each pixel's NDVI value was obtained. To process the data, first we converted each 7-day composite scene to a 14-day composite scene and each 10-day composite scene to a 15-day composite scene by taking the pixel-by-pixel maximum value comparing each scene with its previous scene. This smoothing process has the effect of eliminating all strict local minima (singleton down-spikes) from the raw NDVI time series, as NDVI noise predominantly occurs in the downward direction (Wardlow *et al.* 2006, Brown *et al.* 2013). After computing the 14-day (CONUS) and 15-day (CAM) maximum value NDVI composite time series, we used DOA values to linearly interpolate NDVI to the day (Brown *et al.* 2013). From that approach, we created a regularly spaced, temporally precise, resampled time series with a weekly time step corresponding to days 7, 14, …, 364 (52 samples per year). This concluded the NDVI time series data preparation.

Next, we obtained 10-m land cover data for the monarch sampling location buffers from the European Space Agency (ESA) WorldCover 2021 dataset (https://worldcover2021.esa.int/). Using each 375-m VIIRS pixel as a zone for zonal statistics calculations, land cover fractions were estimated for each VIIRS pixel for the "Grassland" and "Shrubland" classes in the ESA dataset. Monarchs tend to forage on open landscapes, so we discarded all VIIRS pixels within the buffers that were comprised by less than 50% combined grassland plus shrubland. Using the remaining pixels within each monarch sampling location buffer, a spatially averaged NDVI times series was computed to represent each monarch sampling location in subsequent processing.

The final step of the NDVI data preparation involved collapsing the annual NDVI time series to single values guided by the circumstances of the research. Here, we wanted to reflect on nectar availability at the different sampling locations during monarch fall migration. Using a rolling 8-week window and advancing one week at a time, accumulated or time-integrated NDVI values (which provide a proxy for vegetative vigor, and thus presumably also nectar production) were computed for 12 such time windows (collectively spanning 7/26-12/6) by summing the eight NDVI values represented in each

time window. Each site-specific and time-window-specific annualized time series was then converted to a Z-score using sample mean and standard deviation statistics estimated from its 11 years of observations. Lastly, using best professional judgment, the research team determined which time window was most pertinent at each monarch sampling site.

In summary, we are defining an NDVI-based variable that is an integral (or average in this case) across space & across time. With the same land cover metric (≥50% grass/shrub) being used to define a pixel mask inside the 50-km buffer around every site, the variable thus will be unique to each site according to our choice of which integrating time period to use at each site. Because our variable values come from an 11-year NDVI time series covering all of the sites for 2012-2022, we can compute site-specific and variable-specific mean and standard deviation sample statistics, which then allow us to independently convert each of our variables to a Z-score. These Z-scores are instantly relatable without worrying about each site's particular NDVI value characteristics, which vary widely with land cover, latitude, climate, etc. While those details certainly impact monarch activities and outcomes at a location, we seek a measure of locally relative vegetative conditions across time, which is manifested in the Z-scores.

NOTE on NDVI dataset overlap: Comfort (US) and Monterrey (Mex) monarch sample sites have coverage in both the CONUS and the CAM datasets. In these cases, the two Z-score time series (which exhibited good agreement) were averaged point-by-point to create a single Z-score time series.

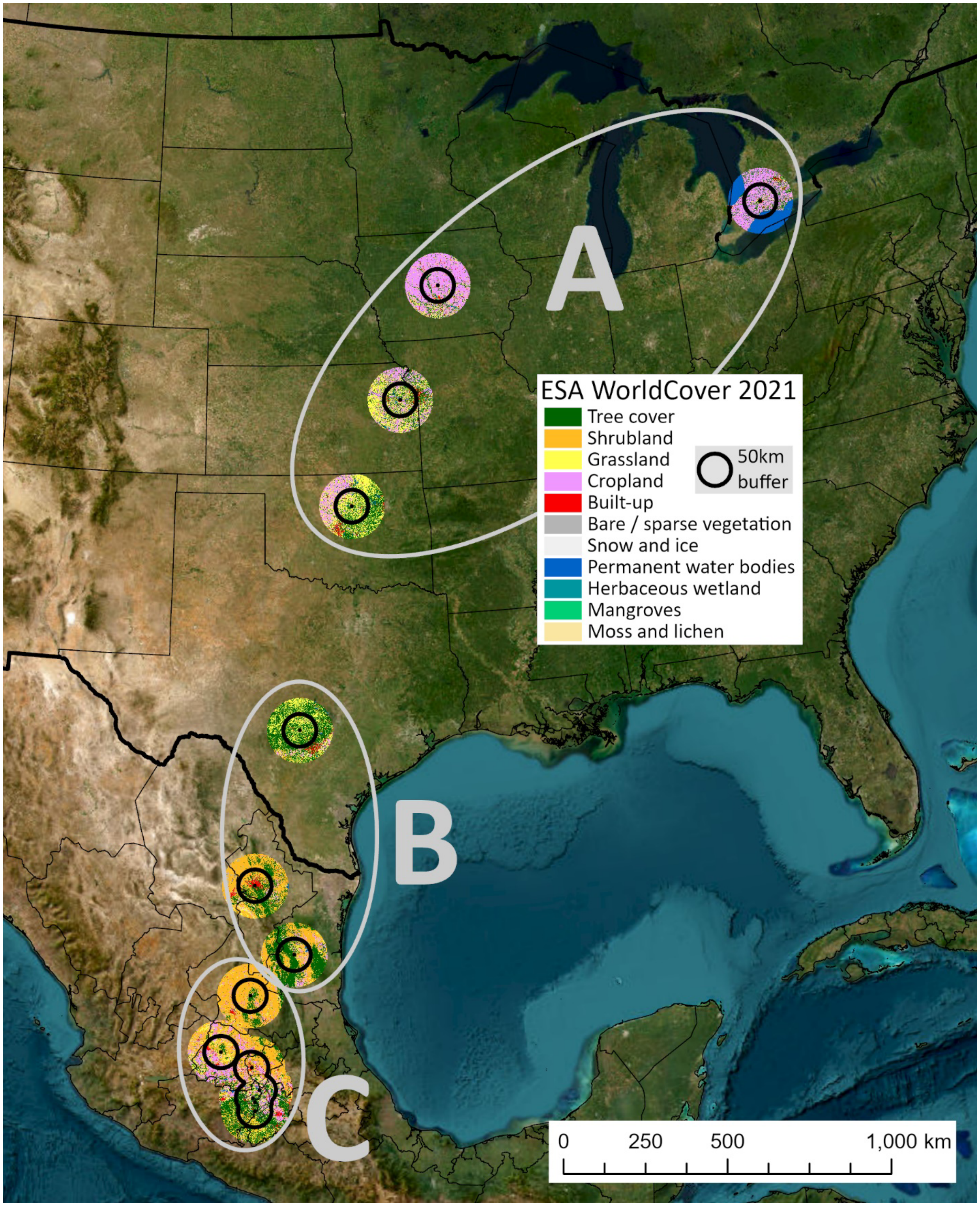

Figure S2. Sample site locations and latitude-elevation groupings (A, B, C; Table S1) are shown, along with land cover data from the ESA WorldCover 2021 dataset used for targeted NDVI signal extraction to create a proxy for local nectaring conditions.

Table S4. Deviations from 11-yr accumulated NDVI measures of greenness (see NDVI Data Preparation) for sample locations are sorted into three elevation groups (Table S1; Figure S2): (A) four locations north of Texas [low elevation, high latitude], (B) Texas and the two northernmost locations in Mexico [medium elevation, medium latitude], and (C) seven locations in the Mexican highlands [high elevation, low latitude]. Sites are ordered by latitude in the table. Z = -0.4, which is nearly equal to the first tercile breakpoint (P[Z < -0.4] = 0.34) in the standard normal distribution, was used as a threshold to identify poor nectar foraging conditions. Red cells fall below the threshold, while yellow cells indicate negative values greater than the threshold. As to the passage of a migration, Group A represents August-September, Group B represents September-October, and Group C represents October-November.

	Group A				Group B			Group C						
	London	Ames	Lawrence	Stillwater	Comfort	Monterrey	Ciudad Victoria	San Luis Potosi	Queretaro	Quanajuato	Contepec	Senguio	Sierra Chincua	Cerro Pelon
	7/29 – 9/16				8/26 – 10/14			9/23 – 11/11						
2019	1.35	0.87	0.55	0.72	-0.43	0.65	-0.55	-0.79	0.61	0.39	1.17	1.57	1.5	0.59
2020	0.4	-1.21	0.69	0.81	0	1.65	1.3	-0.87	-1.58	-1.88	-1.01	-0.8	-0.75	-0.58
2021	1.02	0.08	0.66	0.41	0.14	-0.68	0.24	1.45	0.72	0.59	0.73	0.9	1.43	1.7
	8/5 – 9/23				9/2 – 10/21			9/30 – 11/18						
2019	1.37	1.07	0.61	0.91	-0.58	0.79	-0.57	-0.64	0.66	0.42	1.14	1.44	1.39	0.64
2020	0.39	-1.27	0.66	0.83	-0.01	1.38	0.95	-1	-1.72	-1.89	-1.33	-1.14	-1.12	-0.98
2021	0.61	-0.05	0.62	0.26	0.09	-0.8	0.25	1.25	0.69	0.56	0.61	0.8	1.2	1.57
	8/12 – 9/30				9/9 – 10/28			10/7 – 11/25						
2019	1.21	1.2	0.65	1.07	-0.74	0.84	-0.6	-0.5	0.67	0.43	1.08	1.31	1.26	0.7
2020	0.25	-1.23	0.58	0.77	-0.03	1.15	0.51	-1.17	-1.8	-1.9	-1.61	-1.49	-1.6	-1.58
2021	0.48	-0.16	0.6	0.14	0.08	-0.85	0.19	1.06	0.6	0.5	0.49	0.69	1	1.3
	8/19 – 10/7				9/16 – 11/4			10/14 – 12/2						
2019	1.09	1.27	0.71	1.21	-0.89	0.76	-0.61	-0.31	0.75	0.48	1.1	1.25	1.18	0.79
2020	0.2	-1.18	0.42	0.69	-0.12	0.87	0.04	-1.25	-1.81	-1.87	-1.65	-1.57	-1.7	-1.84
2021	0.5	-0.19	0.6	0.04	0.08	-0.84	0.2	0.88	0.51	0.44	0.36	0.57	0.77	1.02

REFERENCES

Brown JC, Kastens JH, Coutinho AC, Victoria DC, Bishop CR (2013). Classifying Multiyear Agricultural Land Use Data from Mato Grosso Using Time-Series MODIS Vegetation Index Data. *Remote Sens Environ* 130: 39-50. DOI: 10.1016/j.rse.2012.11.009

Taylor OR Jr, Lovett JP, Gibo DL, Weiser EL, Thogmartin WE, Semmens DJ, *et al* (2019) Is the timing, pace and success of the monarch migration associated with sun angle? *Front Ecol Evol* 7:442. doi: 10.3389/fevo.2019.00442

Wardlow BD, Kastens JH, Egbert SL (2006). Using USDA Crop Progress Data and MODIS Time-Series NDVI for Regional-Scale Evaluation of Greenup Onset Date. *Photogram Engin Rem Sens* 72: 1225-1234. DOI: 10.14358/PERS.72.11.1225
